# Clinicians’ perspectives of immersive tools in clinical mental health settings: a systematic scoping review

**DOI:** 10.1186/s12913-024-11481-3

**Published:** 2024-09-18

**Authors:** Jessica Cushnan, Paul McCafferty, Paul Best

**Affiliations:** School of Social Sciences, Education and Social Work, Queen’s University, Belfast, Northern Northern Ireland

**Keywords:** Virtual reality, Immersive, Mental health, Clinicians’, Scoping review, Qualitative

## Abstract

**Background:**

Virtual Reality in mental health treatment has potential to address a wide spectrum of psychological and neurocognitive disorders. Despite the proven benefits, integration into clinical practice faces significant challenges. There is a critical need for research into clinicians’ perceptions of virtual reality due to the gap between rapid technological advancements and their adoption in mental health services.

**Method:**

A scoping review was conducted to comprehensively understand clinicians’ perspectives on the application of immersive virtual reality technologies within mental health settings. 4 data bases were searched, from inception, with the search areas of clinicians’, technology, perspectives and mental health. The scoping review followed the PRISMA-ScR checklist. All results were thematically analysed to identify and categorise themes with a focus on qualitative analyses of clinicians’ experiences and perceptions of VR applications in therapeutic contexts.

**Results:**

17 articles were selected, encompassing a range of mental health settings. The findings indicate that the integration of VR in clinical environments is heavily influenced by clinicians’ knowledge and experience, with unfamiliarity often leading to scepticism. Positive attitudes towards VR, bolstered by direct experience and training, were found to drive acceptance, as clinicians’ acknowledged its potential to complement traditional therapies. However, there are still gaps in understanding VR’s therapeutic applications, particularly concerning its impact on human interaction and its suitability for specific patient groups. Balancing VR’s clinical benefits with ethical and safety concerns is crucial, especially when working with vulnerable populations. Furthermore, structural and administrative support is essential to overcoming the financial and logistical challenges of VR implementation, ensuring its safe and effective integration into mental health care.

**Conclusion:**

While VR holds significant potential for enhancing mental health care, its successful integration into clinical practice necessitates addressing existing gaps in knowledge, training, and structural support. By carefully balancing its clinical benefits with ethical, practical, and safety considerations, VR can be effectively utilised as a valuable tool in mental health treatment, providing innovative solutions while ensuring that patient care remains paramount.

## Background

Immersive technologies, such as Virtual Reality (VR) and game-based interventions, have made significant strides in the field of mental health, demonstrating their potential and promise in therapeutic care [1, 2]. The research surrounding the use of VR, which is defined as a technology that creates a simulated, immersive environment where users can interact with computer-generated sensory experiences, often through visual, auditory, and sometimes tactile feedback. Using headsets or other devices that track movements, users can explore and engage with these virtual spaces in real-time, giving them the sensation of being physically present within the digital world [3]. This capability to design and manipulate these environments is fuelling the growing interest in using VR for both the treatment, and assessment of psychological and neurocognitive conditions, such as anxiety [4], post-traumatic stress disorder (PTSD) [5] obsessive compulsive disorder [6], psychosis [7], children with autism [8], and a range of developmental and learning disabilities [9]. The application of VR offers several key benefits. Firstly, it offers a safe and controlled environment for many of the aforementioned mental health conditions, allowing patients to face their fears within the varied scenarios that VR can provide, whether these are simulated real-life situations or entirely fictional and imaginative environments. This exposure component serves as a tool that has comparable effects, if not more so, than traditional exposure therapies [10]. Due to the immersive and interactive nature, it offers new possibilities to enhance areas of traditional therapy that were previously unattainable [11], for example, the element of personalisation which allows clinicians’ to tailor contextually relevant environments to meet the specific needs of their patients safely [12]. Furthermore, the research has indicated that VR has the potential to open a more engaging and motivational therapeutic platform for patients, which could be especially beneficial for those who are sceptical about therapy or find it challenging and stigmatising [3].

This is particularly relevant given the current state of affairs within the remit of mental health services. In 2019, mental health disorders ranked in the top ten leading causes of burden worldwide, a situation that has shown no reduction since the 1990’s [13]. Moreover, there is a growing treatment gap within mental health care services, seeing an estimated 70% of people who are in need not getting access to them [14, 15].

In addition to the benefits, there is a significant cultural shift in healthcare, reflective of global recognition of the potential of digital solutions. For instance, the World Health Organisation’s Global Strategy on Digital Health [16] sets a foundational tone for this transition, highlighting the need for better training and infrastructure to harness digital technologies to their full potential to enhance healthcare delivery. Furthermore, commitment to enhancing digital competencies are underscored within nationwide government plans such as the Department of Health’s All Ireland Digital Capability Framework [17] and the UK governments Plan for Digital Health and Social Care [18]. This paradigm shift aligns not only with the increasing evidence base of research and technological advancements, but the evolving needs and preferences of service providers and service users [19] opening the doors for a new era for mental health treatment.

To date however, there is an evident gap between the rate in which technology is advancing and the adoption in mental health services [20]. In a recent systematic review, Best and colleagues [21] highlight the implementation difficulties of implementing VR within clinical mental health settings which include perceived costs, the lack of technical standardisations, and low acceptance amongst clinicians’. Additionally, there is broader belief that the technology may impede patient engagement or replace the role of a mental health professional [3]. Currently, there is a gap in the research concerning the detailed qualitative insights of clinicians’ on the use of VR within clinical settings.

## Methods

By adopting the Population, Concept, Context (PCC) Framework to define the research goal and guide the study protocol [22], this review targets clinicians’ (population), the use of immersive VR tools (concept), within the setting of clinical mental health care (context). Therefore, this review aims to combine and analyse evidence from clinicians’ qualitative feedback to answer the question ‘What are clinicians’ views on using immersive VR tools in mental health clinical settings?’ The specific aims were (i) Conduct a scoping review: Following the Joanna Briggs Institute Manual for Evidence Synthesis [22] for Scoping Reviews, research was gathered meticulously to review the clinicians’ viewpoint on the application of VR in mental health settings. This includes studies across various mental health disciplines and clinical environments; (ii) Carry out a thematic analysis and synthesis: A thematic analysis approach was applied to the qualitative data collected using the Braun and Clarke [23] six stage model to allow for the charting of key themes and insights. This method will also allow for the nuanced understanding of the clinicians’ experiences and attitudes towards VR in mental health care; (iii) Report findings and identify research gaps: To identify and report any gaps in the current body of literature. This will include aspects of the VR application in mental health care that may need further investigation or if the clinicians’ perspectives have not been understood or reflected.

### Study design

We utilised an enhanced scoping review methodology, guided by the JBI Manual for Evidence Synthesis [22], which explicitly details each stage of the review process: (1) defining and aligning the objectives and question, (2) developing and aligning the inclusion criteria with the objectives and question, (3) describing the planned approach to evidence searching, selection, data extraction, and presentation of the evidence, (4) searching for the evidence, (5) selecting the evidence, (6) extracting the evidence, (7) analysis of the evidence, (8) presentation of the results, (9) summarising the evidence in relation to the purpose of the review, making conclusions and noting any implications of the findings.

### Search strategy and data sources

After consulting with a subject expert and the subject librarian at Queen’s University, Belfast, we undertook a search across PTSD Pubs, PUB Med, Scopus, and APA PsychInfo databases from October to November 2023, selected for their topic relevance. Initial searches in October 2023 led to further refinement, culminating in a final search in November 2023. We employed Boolean operators and truncation to accommodate different variations of the term “mental,” integrating these terms with ‘AND’ to focus the search further.:

- Clinician OR Therapist.
- VR OR ‘Virtual Reality’ OR VRET OR ‘Virtual Reality Exposure Therapy’.
- Perspective OR View OR Attitude OR experience.
- Mental* OR psychological OR disorder.

All results were imported to EndNote 21 for screening.

### Study selection

To be included in this review, studies needed to meet specific criteria: (1) they must capture the qualitative perspectives of clinicians’ (population), (2) concentrate on the use of immersive VR technology (concept), and (3) be pertinent to mental health care settings (context). The emphasis on VR technology within mental health care aims to directly address the review’s specific interest area. Including studies that offer clinicians’ qualitative insights was deliberately chosen to gather practical and professional perspectives on VR’s application in clinical settings, valuing clinicians’ experiences as key to understanding VR’s real-world impact on mental health care. We opted exclusively for peer-reviewed articles to ensure the reliability and credibility of our findings. Peer review introduces an essential layer of quality control, as these articles undergo rigorous scrutiny before publication [24]. We limited our search to articles in English for practicality, acknowledging potential limitations in scope. While focusing on English-language articles might not encompass all available evidence, this constraint is unlikely to significantly affect our comprehensive findings [25]. We applied no date restrictions, recognising the importance of capturing the full research spectrum on VR in mental health. This approach ensures a broad overview of both historical and recent studies, enriching the review with diverse insights.

**Fig. 1:**
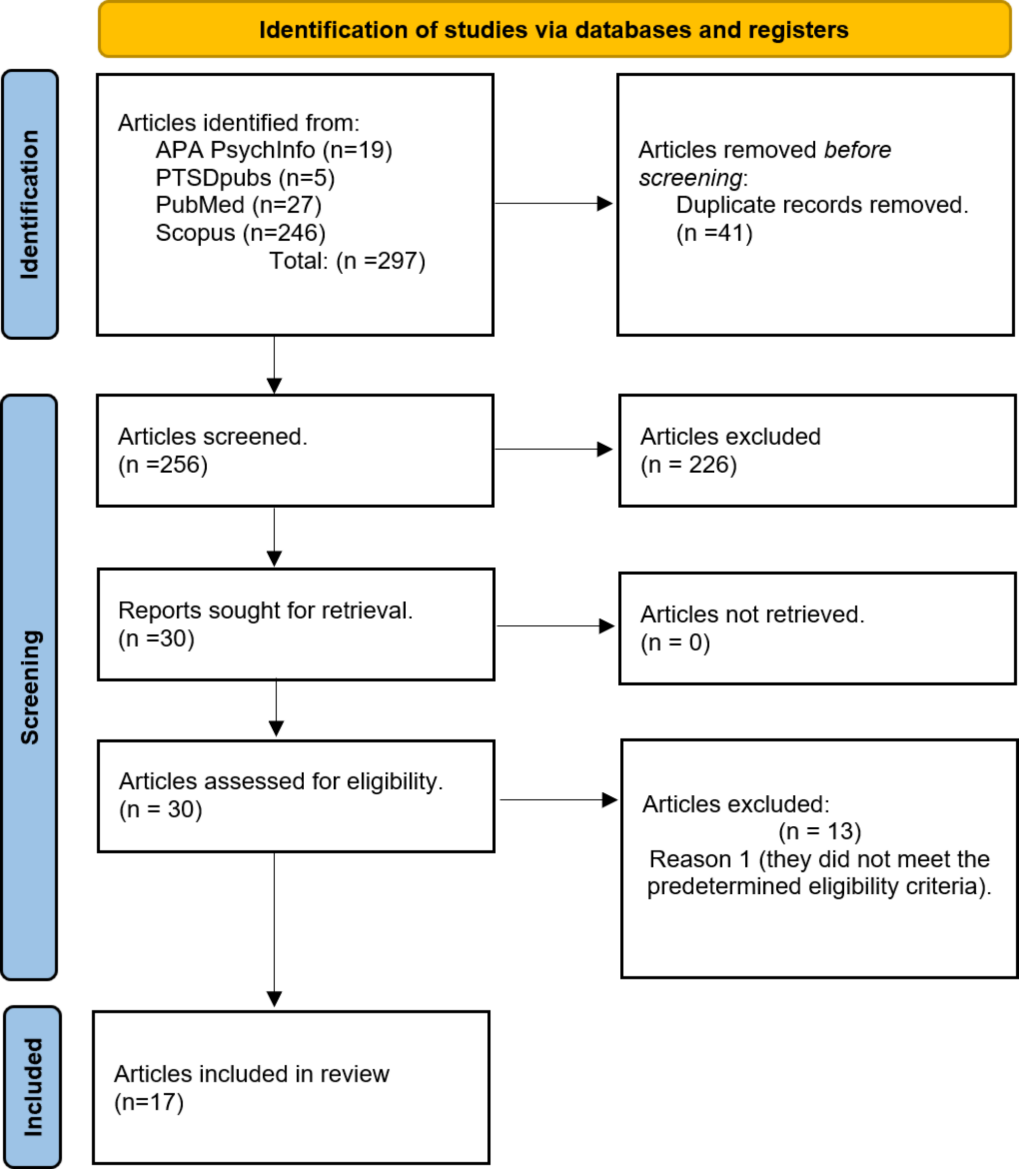
PRISMA 2021 Flow Diagram

In the final phase, the search yield was 297 studies. After removing 41 duplicate records, the remaining 256 articles then underwent an independent screening based on their titles and abstracts in alignment with the predefined inclusion criteria. Each article was then colour coded and categorised as ‘yes’, ‘no’, and ‘maybe’ for further consideration with ‘maybe’ indicating the need for a full text review to determine if the qualitative perspectives of the clinicians’ were included in the data. To uphold the integrity and consistency of the review process a re-evaluation of 100% of articles was conducted by an independent author to ensure inter-rater reliability [22]. This approach led to the preliminary selection of 30 articles for detailed full text review. During the full text phase, any discrepancies between authors were extensively discussed until a unanimous agreement was reached. As a result, a further 13 articles were excluded for not meeting the established criteria, leaving 17 articles deemed relevant and suitable for inclusion in the review. The final step in the search process involved an independent hand search conducted by the primary author among the final 17 articles to identify any additional studies. However, this careful examination did not reveal any new studies for inclusion, solidifying the selection of the final 17 articles. The review selection process is shown in Fig. 1, using the PRISMA diagram [26].

### Quality appraisal

The critical appraisal of the seventeen selected articles, a step beyond the usual scope of scoping reviews, was undertaken to improve the quality of the review. While scoping reviews typically map out research areas without assessing study quality [22], appraising these articles helped to clarify evidence levels and bolster the review’s credibility [27]. This mix of 9 mixed method and 8 qualitative studies underwent appraisal using the JBI Checklist for Qualitative Research [28], following Bryman’s [29] advice for a separate criteria approach for mixed methods. Despite evaluating studies on a ten-point scale, no cutoff was used to exclude studies, aiming for inclusivity and a rich qualitative exploration [30]. To ensure inter-rater reliability, all articles were re-evaluated by an independent author, with discrepancies discussed until consensus, ensuring all seventeen articles were thoroughly and consistently appraised.

### Characteristics and thematic analysis

**Fig. 2:**
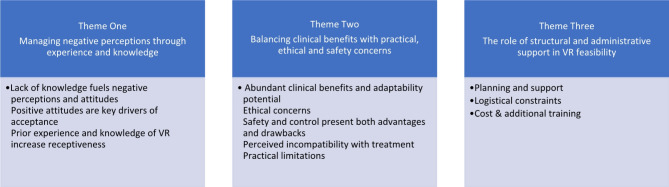
Themes and Subthemes

The characteristics of the included studies are charted in Table 1. Among the 17 studies, 3 were conducted in Australia, United Kingdom, the Netherlands, and Canada. 2 in the United States, and 1 study each in Spain, Germany, and Norway. All of the studies had trained professionals as their participants, however included in this, 2 of the studies also incorporated stakeholders and service managers as participants. The study design included 9 mixed method studies and 8 qualitative studies. This study employed Reflexive Thematic Analysis (RTA) as outlined by Braun and Clarke [23, 31] to analyse the qualitative data collected to explore the perspectives of clinicians’ on the use of immersive VR tools in clinical mental health settings. RTA emphasises the researcher’s active role in identifying and interpreting patterns of meaning within the data, acknowledging that the researcher’s subjective position influences the analysis. This approach is suitable for an inductive analysis, which allowed flexibility in exploring themes within the data for this study. Qualitative data retrieved from the 17 studies was transferred to NVivo 12 Plus analysis software, and the process began with familiarisation, where data was read and re-read to gain a deep understanding. Initial codes were then generated directly from the data without a pre-set framework. These codes were grouped into potential themes that reflected shared meanings. Themes were reviewed and refined to ensure accuracy and coherence. Each theme was then clearly defined and named to capture the core meaning. Finally, the themes were synthesised into a coherent narrative to provide a rich understanding of the findings. These themes and sub-themes are detailed in Fig. 2.

**Table 1:** Characteristics of included studies - data extraction table

Author, Date, Country	Design	Setting and target group	Number of participants	Qualitative Data Collection	Summary of relevant themes
1. Chung et al., 2022 Australia	Mixed Methods	Purposive sampling of clinical and non-clinical staff from three private psychiatric hospitals in Australia.	*N* = 81	Free text in questionnaires.	Three main themes: Acceptability: Technical challenges, artificiality concerns, staff, and patient familiarity issues. Appropriateness: Safety and clinical risk worries, compatibility with treatment philosophies. Feasibility: Logistical, funding, and expertise constraints; equipment and training availability.
2. Chung et al., 2022 Australia	Qualitative	Purposive sample of staff from a private psychiatric hospital offering general and specialist services, with experience in clinical care and operational management.	*N* = 17	Single semi-structured interviews.	Three main themes: Clinical Factors: VR’s perceived effectiveness depends on its ability to engage patients, its therapeutic efficacy across various settings and disorders, and safety/ethical considerations. Organisational Factors: The adoption of VR is influenced by a strong business case, collaborative planning with stakeholders and a service culture open to innovation. Resourcing challenges like cost and space also play a role. Professional Factors: Education and training availability, staff attitudes towards technology, and the VR system’s usability.
3. Chung et al., 2022 Australia	Qualitative	Purposive sample of mental health clinicians and service managers, from a private psychiatric hospital with general and specialist services 18+	*N* = 14	Single semi-structured interviews.	For implementing therapeutic VR Three domains were judged as highly important (beliefs about consequences; environmental context and resources; knowledge), while seven domains were judged as moderately important (social/professional role and identity; emotions; skills; memory, attention, and decision processes; intentions; beliefs about capabilities; social influences).
4. Guillen et al., 2018. Spain.	Mixed Methods	Purposive sample of CBT professionals with over 7 years of experience, working at Clinical Psychology or Emotional Disorders Clinics.	*N* = 10	Open ended opinion sheet.	Clinicians reported VR interventions were well-received by patients and therapists alike, enhancing patient motivation and treatment delivery. VR’s adaptability in exposure therapy was highlighted, offering controlled and customised therapeutic scenarios. While VR was valued for its safety and therapy support, challenges included the absence of standard protocols and ethical concerns. The effectiveness of VR and traditional methods was comparable, suggesting VR’s role as a supportive adjunct in therapy
5. Haeyen et al., 2021 Netherlands	Qualitative	Purposive sample of art and psychomotor therapists across various institutions and organisations in the field.	*N* = 7	Semi-structured interviews & open-ended questionnaires	Clinicians with digital training felt more competent and saw valuable opportunities in integrating digital media into art therapy, despite a cautious approach. The study emphasised the need for preparation and highlighted digital media’s ability to offer unique, engaging experiences not possible in real life, giving clients control and adding active, fun elements to therapy.
6. Haeyen et al., 2021 Netherlands	Qualitative	Purposive sample of art and psychomotor therapists across various institutions and organisations in the field.	*N* = 5	Semi structured interviews & open-ended questionnaires	Therapists see VR as a supplement, not a replacement for traditional arts therapy, appreciating its ability to provide a brief escape and relaxation for clients. They recognise VR’s potential to enhance arts therapy with schema work and experiential techniques, viewing it as a transformative tool that could improve and expand access to psychiatric treatments in a cost-effective manner.
7. Jones et al., 2022 Canada, Netherlands, UK and USA.	Mixed Methods	Purposive sample of English-speaking 3MDR-trained therapists with military backgrounds and experience treating at least one client.	*N* = 3	Single Semi structured interviews & open-ended questionnaires	Findings revealed three key themes: feasibility and function, technical support, and tailored immersion. Therapists suggested improvements for better documentation, data access, and user-friendly software to enhance patient care. Clinicians depended on tech support. Mixed experiences were reported with vendor support, noting a bias towards physical over mental health. A crucial aspect was the need for customisation to address specific patient needs and observations.
8. Jones et al., 2022 Canada, Netherlands, UK and USA.	Qualitative	Purposive sample of 3MDR therapists and key stakeholders, including untrained therapists, all with military backgrounds.	*N* = 18	Semi-structured interviews	Clinicians gained a deeper understanding of client struggles which positively affecting their practice. The immersive, multi-sensory environment helped avoidant patients confront their trauma, enhancing therapeutic outcomes and empowering clients with self-directed therapy, boosting their self-esteem and confidence. This approach also strengthened therapist-client rapport and the therapeutic alliance. Clinicians are optimistic about future, particularly its potential for customisation and tailoring to individual clinical needs.
9. Jones et al., 2022 Canada, Netherlands, UK and USA.	Mixed Methods	Snowball sample of healthcare professionals and first responders to trauma.	*N* = 35	Focus groups & open-ended questionnaires	Key findings emphasise the necessity for the environment to be customised for specific professional and personal needs, reflecting individual traumatic experiences accurately. Participants also showed strong support for its broader application, suggesting its potential benefits extend beyond military personnel to include diverse trauma-affected groups. The adaptability and wide-ranging applicability for treating trauma across various populations were highlighted as areas for further exploration.
10. Kramer et al., 2010. USA	Qualitative	Convenience sample of trained therapists from Veterans Health Administration hospital.	*N* = 16	4 x Focus groups	VR was seen as an advancement in therapy, with clinicians noting its potential despite concerns about realism affecting symptom assessment accuracy and safety issues for veterans with complex trauma. Adoption barriers included clinicians limited technological proficiency and hesitance among older veterans. Resource limitations and concerns about VR affecting the therapist-client relationship, particularly in balancing technology use with client engagement, were highlighted.
11. Mayer et al., 2022. Germany	Mixed methods	Purposive sample of experts with degrees in medicine or psychology, actively working in psychotherapy, psychiatry, or psychosomatics, focusing on claustrophobia.	*N* = 15 therapeutic experts	Single Semi structured interviews	Mixed findings of expert and Patients. 8 themes: feelings and emotions, personal story, telepresence, potential therapeutic effects, barriers, conditions and requirements, future prospects, and realisation. Qualitative interviews revealed that a considerable proportion of participants felt the discrepancy between reality and virtuality. Experts noted that VR intervention might not be suitable for every kind of patient and elderly patients might not benefit from this kind of technology. Some patients could need more individual cues to trigger their anxiety, and therefore, a broader range of intensity levels is needed.
12. Nijman et al., 2020. Netherlands	Mixed Methods	Purposive sample from three Dutch mental health centres, focusing on individuals with psychotic disorders.	*N* = 6 therapist	Open ended questionnaires.	Therapists identified VR’s strengths in facilitating role-play exercises and practicing social scenarios, along with a structured treatment protocol. They generally found the VR software user-friendly and well-supported technically but noted issues with technical reliability and software capabilities. Recommendations included enhancing sound and graphics and introducing new features like environments. Despite these positives, deviations from the protocol and technical problems during sessions were reported.
13. Ose et al., 2019. Norway	Qualitative	Convenience sample of all mental health clinicians from the district psychiatric centre and local mental health service.	*N* = 8	2x focus group interviews	Clinicians saw VR’s potential to tailor training scenarios in a safe environment, predicting it could become crucial in mental health services for applications. They appreciated VR’s ease of initiating training over real-world exposure and noted a positive shift in attitudes towards VR after testing, valuing its role in skill training and safe situational exposure. VR’s ability to simulate severe mental illness experiences was also seen as beneficial for increasing empathy and understanding among caregivers and professionals.
14. Raila et al., 2023. America	Mixed methods	Convenience sample of trained therapists recruited from psychiatric hospital	Not provided	Open ended questionnaires	Clinicians observed that many patients, possibly due to age, struggled with VR technology, necessitating extra training and reminders. Minimal frustration was reported, likely due to immediate clinician support. Despite general unfamiliarity with VR, the structured approach aided treatment but limited emotional processing time, prompting calls for extended VR sessions.
15. Riches et al., 2023 UK	Mixed methods	Convenience sample of assistant psychologists or trainee clinical psychologists from acute and crisis psychiatric services.	*N* = 6 therapists	11 Supervision sessions were recorded and TA applied.	Therapists found VR relaxation enjoyable for patients, increasing engagement despite some triggering imagery. The environments were suitable for high-risk patients, and younger patients found the technology easy to navigate. While therapists provided positive feedback, they noted practical challenges like limited resources, time, staff, and room availability. COVID-19 was initially a concern but is no longer seen as a long-term issue.
16. Sambrooks et al., 2022 UK	Mixed methods	Purposive sample of clinicians experienced in addressing fire-setting behaviours.	*N* = 73	Open ended questionnaires	Clinicians find VR valuable for exposing clients to otherwise unattainable stimuli, enhancing assessments, and increasing motivation. However, they also note challenges like financial constraints, access to technology, potential trauma risks, and resistance from management. Despite these issues, the benefits of VR are generally seen as outweighing the drawbacks.
17. Skeva et al., 2021 UK	Qualitative	Purposive sample of professionals with at least 2 years’ experience in substance use disorders.	*N* = 14	Semi-structured interviews	Participants consistently saw VR as a complementary tool to traditional therapy, effective in symptom management but not in addressing root causes. They recommended integrated therapies to meet diverse recovery needs and warned against premature exposure to triggers in VR. Customising VR cues was suggested to improve realism and engagement, aligning therapy with individual substance abuse patterns. VR was also valued for offering deeper insights into recovery, particularly in family involvement and psychoeducation.

## Results

### Theme one: managing negative perceptions through experience and knowledge

The first, smallest of the three themes, demonstrates how the integration of VR into clinical settings is significantly influenced by how knowledge and experience shape perceptions. A lack of familiarity with VR often fuels negative attitudes and scepticism, creating barriers to its adoption as clinicians’ cited their insufficient skills and experience with VR as a major hurdle [32]. Studies indicate that many clinicians’ have never used VR or are unaware of its therapeutic potential [33], viewing it primarily as a tool for gaming and entertainment [34] rather than a clinical asset. This encompasses an understanding of VR’s applications, objectives, benefits, potential side effects, scenario variety, and follow-up care procedures [35]. This gap in knowledge has the potential to lead to resistance, especially with clinicians’ expressing concerns that VR might be used by private health providers to replace service provision, potentially diminishing human interactions and connections [33]. These worries were founded by the belief that people respond better to the human element, highlighting potential negative workforce attitudes towards technological advances and changes in the work place in general [34, 35]. Furthermore, some clinicians’ have the belief that older veterans may be less inclined to engage in VR-based interventions due to unfamiliarity with the technology [32] and additionally concerns were raised that novice therapists, especially those inexperienced in trauma therapy, may face difficulties in applying complex VR-based treatments [36].

However, positive attitudes towards technology can serve as powerful drivers of acceptance, with clinicians’ recognising the importance of both staff and patient attitudes towards VR [34, 35]. Clinicians’ were generally optimistic about the role of technology in therapy, valuing the new approaches that VR can offer [37], especially given its potential to reach patients who are less engaged with conventional treatments [33]. These positive views are further supported by the cultural popularity of technology and shifts in clinicians’ attitudes [32], perhaps influenced by media portrayals of VR’s potential [38]. Those with VR experience gained valuable insights into its therapeutic applications, as illustrated by their personal experiences with the technology [33]. Training also contributed to more positive attitudes, with clinicians’ recognising VR’s potential in skill training and safe exposure, which helped overcome their initial hesitance [38, 39]. Familiarity with VR’s clinical applications, such as its use for PTSD treatment by Barbara Rothbaum in the USA, further deepened appreciation for its potential [34]. Once clinicians’ saw the benefits of VR as a tool for patient treatment, it increased their willingness to support and promote VR, as well as their understanding of the intervention, leading to more targeted referrals [40].

### Theme two: balancing clinical benefits with practical, ethical and safety concerns

The second, more prominent theme highlighted the significant clinical benefits that VR can offer within mental health services but emphasised the need to carefully balance these advantages against various practical, ethical, and safety concerns. The results affirmed VR’s clinical benefits, with many recognising its potential flexibility to be applied across a wide array of therapeutic areas. VR is recognised as an effective addition to conventional tools, offering realistic practice settings that can expedite therapeutic interventions [33–35]. VR’s role as a complementary tool in enhancing traditional treatments and broadening clinicians’ skills and opportunities was emphasised [37, 41]. Reports indicate that VR interventions are promising and well-received by both clinicians’ and end users [32–35]. Additionally, the effectiveness and user satisfaction of VR, particularly in interactive sessions that include therapist support, were observed [42, 43]. The broad application and potential flexibility of VR were highlighted across various contexts, including social and daily skill training for socially isolated individuals [38], interventions for older adults with hoarding disorder [44], relaxation sessions within psychiatric services [40], and for diverse trauma-affected groups, not just the military [45]. VR has also been effectively tailored for specific uses, such as aiding individuals with fire-setting behaviours [46], and, supporting those recovering from substance abuse by providing recovery insights for family work and psychoeducation [47].

An additional benefit to using VR in therapy is driven by its perceived safety and the ability to provide controlled exposure to various scenarios [33, 38, 41]. VR enhances access and control over therapeutic stimuli [35], allowing therapists to monitor sessions and intervene as needed, thereby ensuring client safety [39]. This safety contributes to clients feeling more secure [37] and the structured environment of VR empowers clients by providing a safer alternative to real-life exposure [36], which is particularly beneficial in managing high-risk behaviours [47, 48].

However, despite the benefits, this perception of safety is counterbalanced by significant concerns about the potential clinical risks and ethical dilemmas posed by VR, particularly the fear that it could exacerbate symptoms or lead to avoidance of real-world interactions if not used correctly. Concerns were raised about using VR without proper training or thorough client assessments, which could increase the risk of negative outcomes [33]. While some clinicians’ view these risks as comparable to traditional methods, others advocate for stringent protocols to ensure safe and ethical use, particularly to avoid triggering content [35]. Ethical considerations also include maintaining professional boundaries to prevent injury [34]. Additional concerns involve the potential for VR to worsen symptoms in vulnerable populations, such as veterans, and the risk of re-traumatisation or triggering trauma, which stresses the need for careful patient selection and management [32, 46, 47].

Concerns about the compatibility of VR with certain therapeutic philosophies also present challenges to its adoption. Some professionals are sceptical about VR’s fit with therapeutic approaches that emphasise creativity and strength-based methods, such as art therapy [33]. Clinicians’ from non-cognitive-behavioural backgrounds, particularly those practicing psychodynamic therapy, question VR’s effectiveness in addressing the underlying causes of anxiety, as it tends to focus on behavioural symptoms instead [42]. Concerns have also been raised about VR’s interaction with psychotropic medications and its potential to alter patients’ perceptions or exacerbate detachment [35]. Additionally, the appropriateness of VR for severe conditions like schizophrenia, and its challenges for individuals sensitive to VR’s sensory inputs, such as those with vision impairments, are significant concerns [32, 34, 39, 45]. While VR offers substantial clinical benefits and therapeutic potential, these advantages must be carefully balanced with the legitimate, ethical and safety concerns associated with its use in mental health services.

Finally, clinicians’ raised practical concerns about equipment reliability, the need for software customisation, and the user-friendliness of the VR technology [33, 35, 42]. Issues such as the bulkiness of headsets, the lack of validated scenarios, and limitations in the realism and personalisation of VR environments stand out as main issues [32, 34, 47]. Additionally, the complexity of using VR for certain patient groups, particularly the elderly who often require additional training [43, 44], has left clinicians’ at times struggling to effectively deliver interventions to patients who lack technological proficiency [40].

### Theme three: the role of structural and administrative support in VR feasibility

The final theme examines the broader logistical elements and the role of structural and administrative support, highlighting how these factors are crucial in determining the feasibility of implementing VR in clinical settings. The time required for VR interventions often exceeded initial estimates [43], which poses a challenge in busy psychiatric environments where clinicians’ primarily work in groups, VR was perceived as an isolating activity, creating practical difficulties due to workload and time constraints [33], with the additional consideration that a separate room might even be needed [42]. Clinicians’ also raised concerns about access to appropriate resources and technology to address the disconnect and relationship between clinicians’ and patients during VR sessions, such as reliable Wi-Fi, and the necessity for preparatory measures like additional TV screens or mobile devices to stream and monitor client activities [32, 39, 40, 46]. It was evident, however, that there was shared confidence among clinicians’ that with the right support and forward planning, challenges such as funding, space, time, and resources could be overcome [33, 39]. Support from managers was considered crucial, as they played a significant role due to their influence over other staff members and general service operations [34]. In studies where VR interventions were successfully implemented, the quality and availability of technical support were highly praised [36, 43], with clinicians’ at all sites feeling they had adequate support to manage in-session situations. Furthermore, clinicians’ generally felt they were able to receive support from other professionals using the technologies globally, highlighting the wider support network available [48].

Cost and the facilitation of additional training were also flagged as prominent concerns, due to the notable lack of expertise to provide adequate training for clinicians’ [35]. Managers reported that they need expert advice on the evidence base, available hardware and software, training resources, and implementation strategies [35, 45]. Clinicians’ unanimously questioned the impact of previous investment in quality improvement activities, which could constrain resources or affect organisational stability. This concern was compounded by the additional costs associated with purchasing and maintaining VR, providing staff training, and ongoing technical assistance [32, 35, 46]. The financial viability of VR was also questioned, with participants perceiving that VR might not be viewed as financially lucrative enough by private stakeholders [33, 34]. However, it was argued that this fiscal barrier might be lessened if the VR program was suitable for other clinical applications, making it more cost-effective and useful for a broader population [32, 42, 45, 46].

As mentioned in the second theme, there are identified gaps in procedural knowledge, including how to select appropriate clients for VR, apply VR clinically, and manage safety risks and procedures [34], with the need for technology to be simplified for easy use [41]. This highlights the importance of access to treatment manuals, in-service training days, the development of clinical governance processes, and consultation opportunities with VR developers and early adopter services [33]. There were mixed responses to the manualised nature of a VR protocol, some clinicians’ found it helpful in guiding treatment implementation and considered it intuitive and easy to use, while others felt it limited therapy time, with deviations from the protocol being reported [40, 43, 44]. Additionally, similar to the first theme of managing negative perceptions through experience and knowledge, after applying guided VR interventions, clinicians’ were willing to participate in future studies or adapt their clinical practice to include this novel intervention [36]. Clinicians’ became more confident about using VR in mental health care after testing it themselves and recognised opportunities for using VR in different situations [38]. Interestingly, not all clinicians’ deemed formal training and protocols necessary, suggesting that simply being given a platform within a clinical setting to explore VR with the patient could enhance engagement [37].

## Discussion

### Managing negative perceptions through experience and knowledge

While this study focused specifically on the use of VR in clinical settings, it is important to acknowledge the growing global trends in research, that explore the use of diverse technologies in the field of mental health [49]. This is perhaps reflected in the results of the first theme, which revealed considerable positivity and openness among clinicians’ towards integrating technology into their practice. Positive attitudes and perceptions highlight optimism about the future role of VR in therapy, particularly its potential to complement conventional therapies by offering novel approaches when other methods are less effective [50, 51]. This move towards more technology-centric approaches is supported by research, showing that attitudes towards VR exposure therapy are no longer a barrier to its implementation [52] reflecting a cultural shift towards embracing VR within clinical settings.

There were, however, definite gaps in understanding VR’s therapeutic applications which generated certain negative attitudes and scepticisms on behalf of some clinicians’. These attitudes were likely not due to indifference but rather a lack of knowledge about the clinical applications of VR. Prior experience and familiarity with VR play a crucial role in shaping its perceived therapeutic potential as clinicians’ with direct experience in using VR in clinical settings tend to have a deeper appreciation of its benefits [53, 54]. Research consistently shows that education and training are key to enhancing clinicians’ competence and willingness to integrate VR into their practice, helping to overcome initial hesitance [55, 56].

Apprehensions that VR could replace traditional service provision and reduce human interaction were raised. Research emphasises that the clinician-patient relationship remains paramount when using VR interventions, which should complement rather than replace human empathy and support in therapy [57]. While there is a perception that vulnerable patients, particularly those seeking mental health treatments, may prefer human connections [58], some studies have found that some patients actually prefer using VR over traditional methods [11, 59]. Additionally, recent studies like THRIVE—a four-session automated cognitive VR intervention—demonstrate that automated VR can be effective and offer advantages in cost efficiency and treatment accessibility [60]. However, due to the automation, it also carries risks of reduced human interaction. These findings highlight the need for further research to investigate service users’ preferences through patient and public involvement, addressing the apprehensions surrounding reduced human interaction [61].

As clinicians’ gain more experience and knowledge, the growing acceptance and optimism towards integrating VR into clinical practice also bring to light concerns about the practical, ethical, and safety implications of this technology. These concerns form the basis of the second discussion section, which explores the need to balance the undeniable clinical benefits of VR with the challenges posed by its implementation. As enthusiasm for VR continues to rise, it becomes increasingly important to critically evaluate how these tools can be safely and effectively integrated into practice, ensuring they enhance, rather than detract from, patient care.

### Balancing clinical benefits with practical, ethical and safety concerns

The primary therapeutic component of VR is widely recognised to be its exposure element [1]. While VR technology offers the potential to enhance patient safety, control, and adaptability across various interventions, these features also raise concerns. Firstly, even with potentially triggering imagery, [3, 5]. VR exposure has proven effective in increasing engagement. The controlled environment of VR provides a unique opportunity for safe exposure to various situations, allowing clinicians’ to monitor and intervene as needed [62–65]. This not only offers a safety net for patients but also empowers them with a sense of control and security that may not be achievable through traditional therapeutic methods. Additionally, the capacity for VR to simulate relevant stimuli for high-risk behaviours without exposing clients to actual dangers highlights its value as a therapeutic tool [46, 47]. However, clinicians’ express concerns about using VR with vulnerable patient groups, fearing the potential for re-traumatisation, symptom exacerbation, or avoidance of real-world interactions. Despite these concerns, research has shown positive outcomes in using VR with populations such as those with dementia [66], schizophrenia and psychosis [53], Autism Spectrum Disorder [67, 68], and Attention Deficit Hyperactivity Disorder [69]. Additionally, there were apprehensions around the suitability of VR for older patients. However, this concern may be based on outdated stereotypes or as highlighted in the first theme, negative perceptions stemming from a lack of knowledge and experience with VR. Recent research specifically focusing on older adults with mental health issues challenges the assumption that older individuals cannot benefit from or are intimidated by VR technology. Studies have reported promising outcomes, demonstrating that older adults can indeed engage with and benefit from VR interventions [70–72].

Given the unique approach that VR offers through its immersive environments, the realism and customisation capabilities of VR equipment and software are sometimes perceived as limitations, particularly when they are not identical to the real world. However, evidence suggests that VR does not need to perfectly mimic reality to be effective for therapeutic purposes. Studies have shown that VR can successfully elicit the therapeutic responses necessary for psychological treatment, even without perfect realism [73–75]. In addition, one effective study demonstrated that VR was simply used to set the scene, enabling patients to engage in their own imagery exercises within the VR environment thereafter [76]. This is particularly significant considering that highly customisable VR experiences might not always be accessible for standard practice or may not yet be developed for specific types of traumas. Furthermore, a meta-analysis of attrition rates in VR exposure therapy for anxiety-related disorders found that drop-out rates were comparable to those for traditional in vivo exposure [77]. This highlights VR’s effectiveness, suggesting that if it were not a viable treatment option, we would likely see a higher rate of dropouts in comparison to traditional methods.

The juxtaposition between the potential benefits and practical challenges of using VR is a recurring theme in the literature [73, 78–80]. This raises the question: do the clear clinical benefits of VR outweigh, or at least balance, the practical, ethical, and safety concerns raised by clinicians’? Reflecting on the sentiments by the pioneers of technology and mental health, Bouchard and Rizzo [81], it is crucial for clinicians’ to approach the use of VR with discernment, rather than adopting the technology simply because it is available. Essentially then, giving clinicians’ the autonomy to use VR as an additional tool to expedite interventions or as an adjunct to traditional therapies, particularly when other treatment options have been exhausted [50, 51], would enable the development of flexible, tailored treatment plans. This approach would allow for clinicians’ to make informed decisions based on practical, ethical, and safety concerns, ensuring that the treatment aligns with the specific needs of their patients.

Nonetheless, to allow clinicians’ the flexibility to use VR within their practice, the ongoing concerns reiterated in the literature regarding the scarcity of empirically supported scenarios and evidence-backed VR programs, as highlighted by Bell et al. [3] must be addressed. Providing clinicians’ with robust evidence and guidelines will enable careful screening and preparation before integrating VR into therapy. The need for specific protocols to ensure the safe and ethical use of VR is further emphasised by Best et al. [21], who, in a systematic review, noted the lack of detailed clinical guidelines for VR applications. Moreover, integrating VR into existing treatment frameworks presents challenges, particularly outside of Cognitive Behavioural Therapy (CBT). While VR aligns well with CBT’s focus on behavioural modification, its compatibility with psychodynamic approaches, which explore deeper psychological processes, remains underexplored. Addressing these concerns requires the development of detailed clinical protocols, ethical guidelines, and comprehensive training programs, which will be further explored in the third and final section.

### The role of structural and administrative support in VR feasibility

The role of structural and administrative support is closely intertwined with the previous discussion sections, particularly when it comes to the cost and training challenges associated with VR implementation in mental health settings. Historically, the high costs of acquiring and maintaining VR equipment have been a significant barrier [54, 82, 83]. Moreover, the need for comprehensive staff training, which incurs additional expenses, creates a cycle that hinders broader implementation, as noted in theme one, the lack of training and education around VR significantly limits the uptake of VR.

Clinicians’ and managers widely acknowledge that hesitancy towards adopting VR often stems from concerns over costs and the necessary training [84]. This creates a paradox, as integrating VR into practice could lead to more efficient use of clinician time and enhanced treatment outcomes, potentially resulting in cost savings. Importantly, the costs of VR equipment have reduced significantly, making it more accessible and affordable for healthcare providers [21, 83], however, despite these reductions, many studies still highlight cost as a significant barrier [85, 86]. These costs, however, could be mitigated if VR is utilised for multiple applications within therapeutic settings, enhancing its overall cost-effectiveness. Additionally, recent research is offering low-cost, viable solutions [87, 88], and demonstrating the potential of using automated VR in psychiatric care to reduce the strain on already stretched services [60, 89].

Health and Social Care (HSC) systems are increasingly adopting digital skills strategies, like the Topol Review [90] and the All-Ireland Digital Capability Framework [17], to enhance service delivery and workforce digital literacy. While training may suffice for small, incremental changes in digital tool adoption, more significant shifts require substantial structural adjustments [91]. The implementation of VR in clinical settings, though promising, has received mixed feedback, particularly regarding its manualised protocols. Ensuring patient safety is crucial, but flexibility is also needed for patient-centred care. Addressing challenges related to funding, space, and resources through strategic planning is essential. Expert guidance on evidence-based practices, technology options, and implementation strategies is vital [92]. Successful VR interventions are supported by strong technical support and collaborative efforts, highlighting the importance of global networks [93]. Ultimately, the feasibility of VR in clinical settings hinges on overcoming technological, logistical, and financial barriers while maximising the benefits through coordinated training, education, and planning.

### Limitations

This scoping review has limitations, notably its exclusion of grey literature and restriction to English-language articles, potentially overlooking significant research on VR in mental health conducted worldwide. The search across four databases might have missed studies in other databases such as CINAHL and APA PsycNet, and despite a rigorous review protocol, bias cannot be completely eliminated. Additionally, not imposing date restrictions to the search strategy provided an extensive literature overview, offering a broad view of the evidence.

## Conclusion

The integration of VR into clinical settings represents a promising yet complex evolution in mental health care. While there is growing acceptance and optimism among clinicians’ about incorporating VR into practice, significant challenges remain. These challenges are deeply interconnected with the need for robust structural and administrative support, as well as the practical, ethical, and safety considerations that must be addressed to ensure VR’s effective and safe use. Empowering clinicians’ to use VR as an additional tool in therapy involves addressing these challenges through comprehensive training, strategic planning, and the development of evidence-based guidelines. Additionally, the findings highlight the need for further research to investigate service users’ preferences, particularly through patient and public involvement. This research is essential to address apprehensions surrounding reduced human interaction, the suitability of VR for vulnerable user groups, and the overall adaptability of VR across various conditions and demographics. As advancements in VR technology continue to unfold, these investigations will be crucial in ensuring that VR is used in a manner that maximises its benefits while safeguarding patient welfare and enhancing therapeutic outcomes.

## Data Availability

No datasets were generated or analysed during the current study.

## Abbreviations

VR: Virtual Reality
PTSD: Post Traumatic Stress Disorder
CBT: Cognitive Behavioural Therapy
PCC: Population, Concept, Context
JBI: Joanna Briggs Institute
HSC: Health and Social Care
